# Metastatic to negative lymph node ratio demonstrates significant prognostic value in patients with esophageal squamous cell carcinoma after esophagectomy

**DOI:** 10.18632/oncotarget.19673

**Published:** 2017-07-28

**Authors:** Xiao-Feng Duan, Peng Tang, Xiao-Bin Shang, Hong-Jing Jiang, Zhen-Tao Yu

**Affiliations:** ^1^ Department of Esophageal Cancer, Tianjin Medical University Cancer Institute and Hospital, National Clinical Research Center of Cancer, Key Laboratory of Cancer Prevention and Therapy of Tianjin City, Clinical Research Center for Cancer of Tianjin City, Tianjin, China

**Keywords:** esophageal squamous cell carcinoma, esophagectomy, lymph node ratio, MNLNR, prognosis

## Abstract

**Aims:**

The prognostic value of metastatic lymph node ratio (LNR) has been reported in some studies; however, there is no report on the prognostic significance of metastatic to negative lymph node ratio (MNLNR) in cancer patients. The aim of this study was to compare the prognostic value of pN, LNR and MNLNR on the survival of patients with esophageal squamous cell carcinoma (ESCC) after esophagectomy.

**Methods:**

The data of 290 patients with ESCC after curative esophagectomy was retrospectively analyzed. The optimal cut-point for LNR and MNLNR were set as 0, 01-0.2, and >0.2. Univariate and multivariate analyses were performed to identify prognostic factors for overall survival (OS).

**Results:**

Patients classified as LNR 0, 0.01-0.20, and 0.21-1.0, the observed 5-year OS rates were 46.6%, 26.0%, and 11.6%, respectively (*P* = 0.000). Patients classified as MNLNR 0, 0.01-0.20, and >0.2, the observed 5-year OS rates were 46.6%, 31.2%, and 7.4%, respectively, respectively (*P* = 0.000). The pN stage, LNR or MNLNR category was confirmed as a significant independent prognostic factor, respectively (*P* = 0.032, *P* = 0.011 and *P* = 0.003, respectively); However, only the MNLNR category (*P* = 0.003) remained as a significant prognostic factor when the pN stage, LNR and MNLNR category simultaneously included in the multivariate analysis models.

**Conclusions:**

The MNLNR was recognized as an independent prognostic factor in ESCC patients after curative esophagectomy. In addition, MNLNR showed better prognostic value than pN stage and LNR category.

## INTRODUCTION

Esophageal cancer (EC) is the eighth most common cancer and the sixth most common cause of cancer-related deaths worldwide [1]. In 2012, there were an estimated 455,800 new EC cases and 400,200 associated deaths worldwide [1]. In China, there were an estimated 286,700 new EC cases and 210,900 associated deaths [2]. The prognosis of patients with EC remains poor with 5-year OS rates of 15% - 25% for all EC patients [3]. The two main types of EC are squamous cell carcinoma and adenocarcinoma. In high-risk area, including China, esophageal squamous cell carcinoma (ESCC) accounts for > 90% of all EC cases [1]. Surgical resection is still the main treatment option for ESCC patients. Despite a marked reduction in surgical morbidity and mortality and the application of comprehensive treatment, the long-term survival of esophageal cancer patients after surgery remains poor. This poor prognosis has prompted us to investigate more effective prognostic factors for long-term survival.

Lymph node metastasis is an important prognostic factor and indicator for subsequent adjuvant treatments in EC patients. However, a small number of removed lymph nodes (LNs) may lead to under-staging and subsequent under-estimation of disease severity, which is referred to as stage migration [4]. Lymph node ratio (LNR), which is calculated as the ratio of the number of metastatic LNs to the total number of removed LNs, has been proposed to address the problems related to the variability of nodal examination. It has been confirmed as a highly reliable indicator used to evaluate the prognosis of cancer patients. The advantage of LNR over the 7^th^ American Joint Committee on Cancer (AJCC) N category in predicting the prognosis of ESCC patients has been explored in some studies [5–15].

A recent study also showed that the number of negative lymph nodes (NLNs) could impact the OS of patients with ESCC, especially among those with nodal-positive disease and advanced T-stage tumor [16]. The prognostic value of NLNs was also confirmed in previous studies that revealed that a higher number of NLNs was associated with better OS in EC patients after esophagectomy [17–18]. Metastatic to NLNs ratio (MNLNR), which is calculated as the ratio of the number of metastatic LNs to the NLNs, has not been proposed to evaluate its prognostic value in any cancer patients after curative resection. In the light of these considerations, our study was conducted to 1) show whether MNLNR could address the problems related to the variability of nodal examination and stage migration, 2) determine the prognostic value and the relation of MNLNR with OS, 3) compare MNLNR with pN stage and LNR for the prognostic evaluation of ESCC after curative esophagectomy.

## RESULTS

### Patient demographics

The clinicopathological parameters of the 290 ESCC patients included in our study were summarized in Table 1. There were 238 males and 52 females with a median age of 68 years (range, 35-95 years). According to histological grade, 19 patients were well differentiated, 240 were moderately differentiated, and 31 were poorly differentiated. In the cohort, the average number of total retrieved LNs per patient was 15.2 (range, 4-58). According to the 7th edition AJCC TNM staging system, 172, 71, 30, and 10 patients were classified as N0, N1, N2, and N3, respectively. As regard to the TNM staging system, 17 patients were in stage I, 104 patients were in stage II, 155 patients were in stage III, and seven patients were in stage IV.

**Table 1:** Clinicopathological features and univariate survival analysis

Variables	n = 290	5-year OS (%)	HR	95% CI	χ2	P
Gender			0.595	0.358-0.988	4.205	0.040
Male	238	35.2				
Female	52	48.6				
Age (years)			1.326	0.958-1.836	2.981	0.084
< 65	118	46.5				
≥ 65	172	31.3				
Smokig history			1.118	0.792-1.579	0.417	0.519
Yes	208	35.6				
No	82	41.5				
Drinking history			1.109	0.087-1.552	0.418	0.518
Yes	146	36.2				
No	144	38.8				
Tumor location			1.186	0.830-1.696	4.642	0.098
Upper	15	57.8				
Middle	220	34.8				
Lower	55	37.9				
Tumor size			1.007	0.712-1.446	0.022	0.966
< 4cm	174	38.5				
≥ 4cm	116	33.8				
Differentiation			0.725	0.525-0.098	4.060	0.044
Well	19	56.0				
Moderate	240	38.0				
Poor	31	24.9				
pT stage			1.185	1.022-1.374	5.272	0.022
T1-2	61	43.4				
T3-4	229	36.0				
pN stage*			1.493	1.201-1.856	7.372	0.007
0	172	46.6				
1	71	41.1				
2	30	22.1				
3	10	0				
pTNM stage*			1.578	1.202-2.073	19.050	0.000
I	17	63.2				
II	104	42.7				
III	155	32.4				
IV	7	0				
LNR*			1.451	1.223-1.721	16.158	0.000
0	172	46.6				
∼ 0.2	72	26.0				
>0.2	39	11.6				
MNLNR*			1.601	1.265-2.025	17.375	0.000
0	172	46.6				
∼ 0.2	65	31.2				
>0.2	46	7.4				

* Seven patients can not evaluate the N status; the total number of patients was 283.

Log-rank test was used to compare survival in groups. LNR, metastatic lymph node ratio; MNLNR, metastatic/negative lymph node ratio; OS, overall survival; HR, hazard ratio; CI, confidence interval.

### Correlation of the number of retrieved nodes to metastatic nodes, LNR and MNLNR

**Figure 1:**
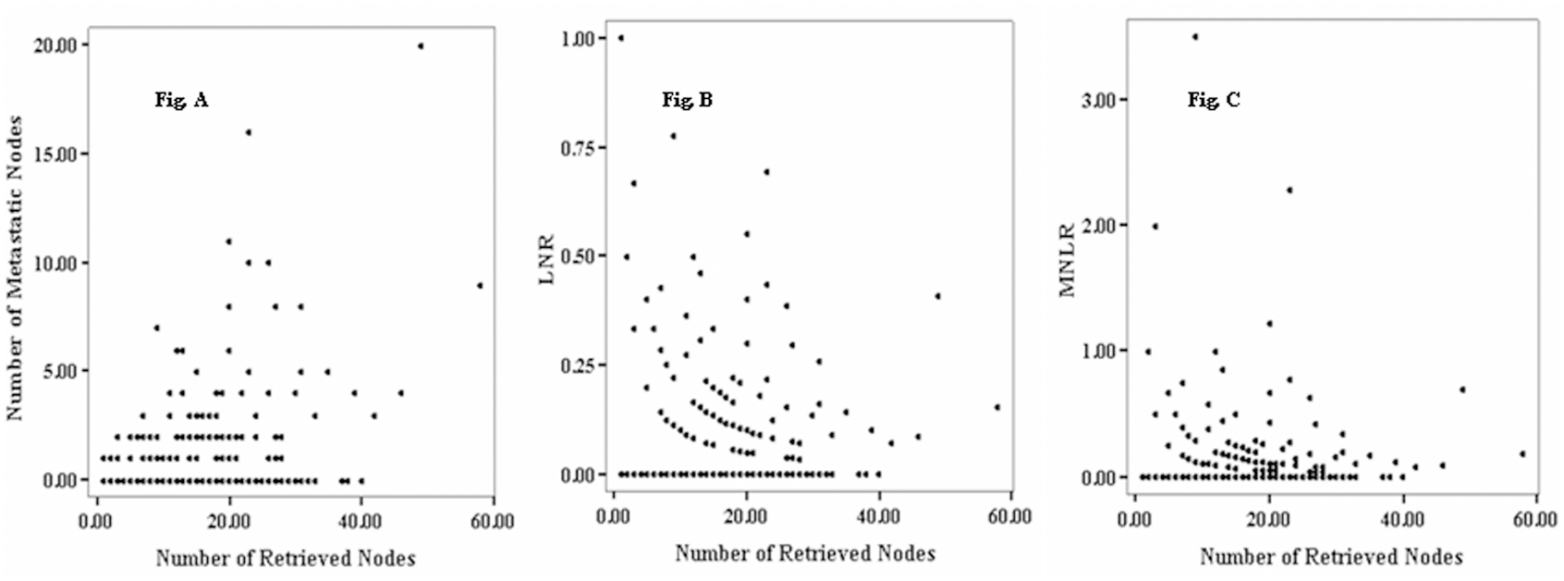
Spearman correlation of the number of retrieved nodes, metastatic nodes, LNR, and MNLNR **(A)** Significant correlation of the number of metastatic nodes with the total number of retrieved lymph nodes (r = 0.168, P = 0.005). **(B** and **C)** No significant correlation of the LNR (r = 0.041, P = 0.491) and MNLNR (r = 0.068, P = 0.254) with the number of retrieved lymph nodes.

Spearman’s correlation analysis showed that the total number of retrieved LNs was signifcantly related to the number of metastatic LNs (r = 0.168, *P* = 0.005; Figure 1A), whereas the number of retrieved LNs was not correlated with LNR (r = 0.041, *P* = 0.491; Figure 1B) and MNLNR (r = 0.068, P = 0.254; Figure 1C).

### The optimal cut-point value of LNR and MLNR

The patients without lymph node metastasis were classifed as LNR 0. Furthermore, the other patients were stratified into five groups by every 0.20 interval of LNR. There were only 13 patients with a LNR value of 0.41-1, and the 13 patients were grouped as > 0.4. According to the best cut-off approach by the log-rank test, the survival rates for the categories 0.21-0.4 and > 0.4 were similar. So we divided the LNR into three subgroups as follows: LNR, 0 (n = 172); LNR, 0.01-0.2 (n = 72); LNR, >0.2 (n = 39).

The patients without lymph node metastasis were classifed as MNLNR 0. Furthermore, the other patients were stratified into five groups by every 0.20 interval of MNLNR. There were only 23 patients with a MNLNR value of >0.4, and the 23 patients were grouped as > 0.4. According to the best cut-off approach by the log-rank test, the survival rates for the categories 0.21-0.4 and > 0.4 were similar. So we divided the MNLNR into three subgroups as follows: MNLNR, 0 (n = 172); MNLNR, 0.01-0.2 (n = 65); MNLNR, > 0.2 (n = 46).

### Univariate survival analysis

The 1, 3, 5-year OS rates of the entire cohort were 81.0%, 62.0% and 37.3%, respectively. For patients classified as N0, N1, N2, and N3 according to the AJCC N category, the 5-year OS rates were 46.6%, 41.1%, 22.1% and 0, respectively (χ2 = 7.372, *P* = 0.007 by log-rank test).

Patients classified as LNR 0, LNR 0.01-0.2, and LNR 0.21-1, the observed 5-year OS rates were 46.6%, 26.0%, and 11.6%, respectively, and the median survival times of these three groups were 58.0, 36.0, and 24.0 months, respectively (χ2 = 16.158, *P* = 0.000 by log-rank test).

**Figure 2:**
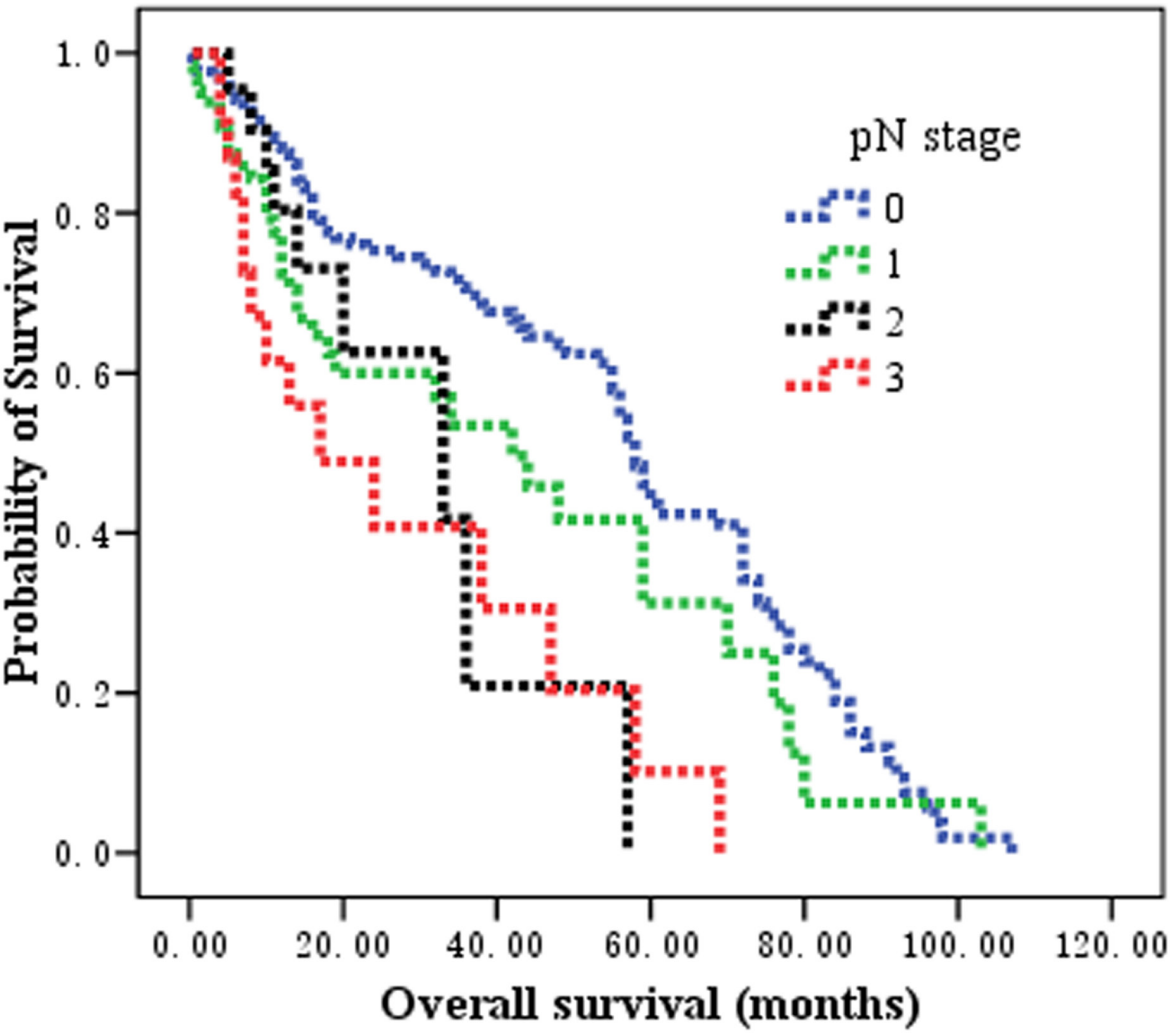
The prognostic significance of pN stage on overall survival in patients with esophageal squamous cell carcinoma after esophagectomy Kaplan-Meier survival analysis of pN subgroups by log-rank test (Chi-square = 7.372, *P* = 0.007).

**Figure 3:**
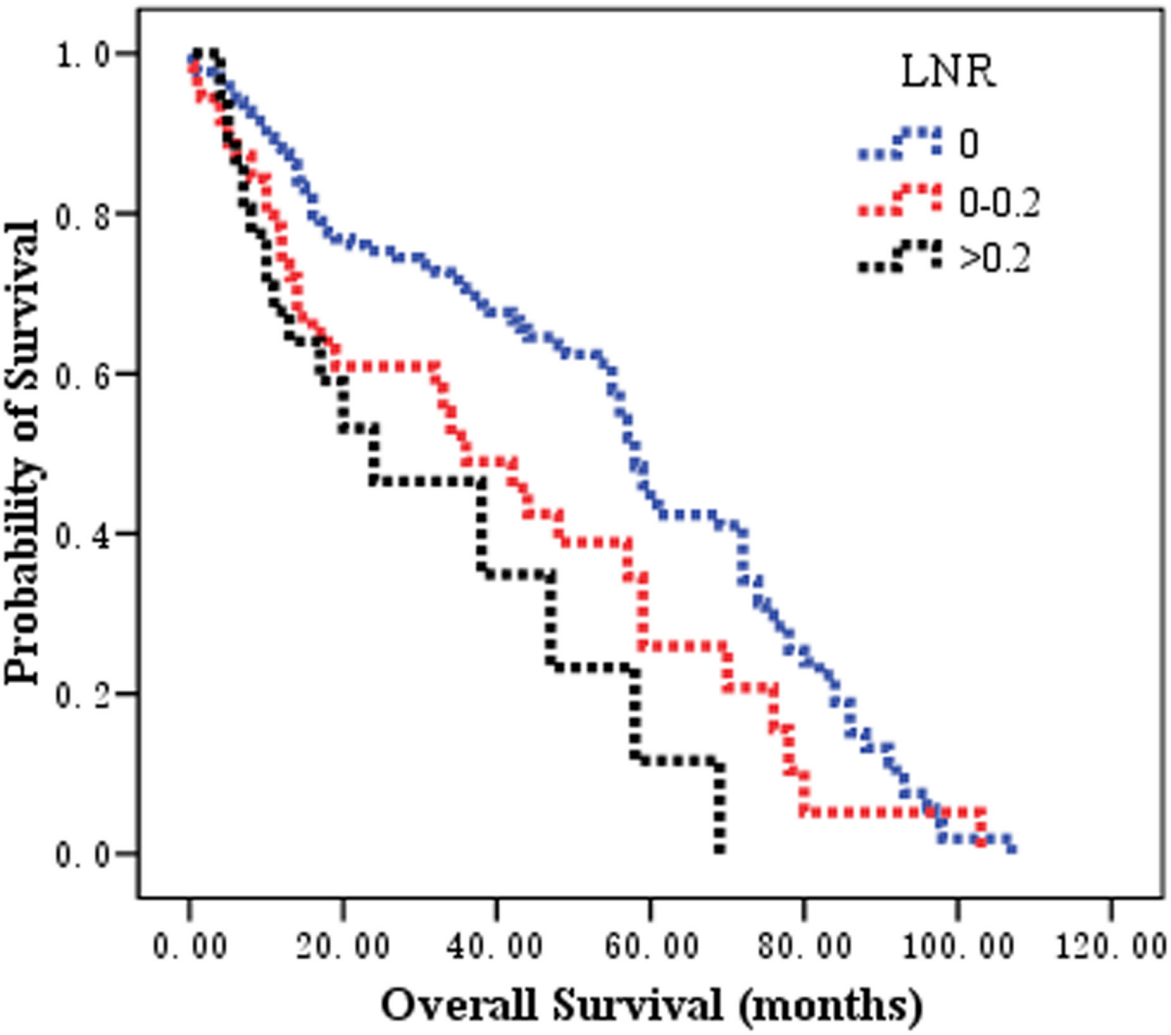
The prognostic significance of LNR category on overall survival in patients with esophageal squamous cell carcinoma after esophagectomy Kaplan-Meier survival analysis of LNR subgroups by log-rank test (Chi-square = 16.158, *P* = 0.000).

**Figure 4:**
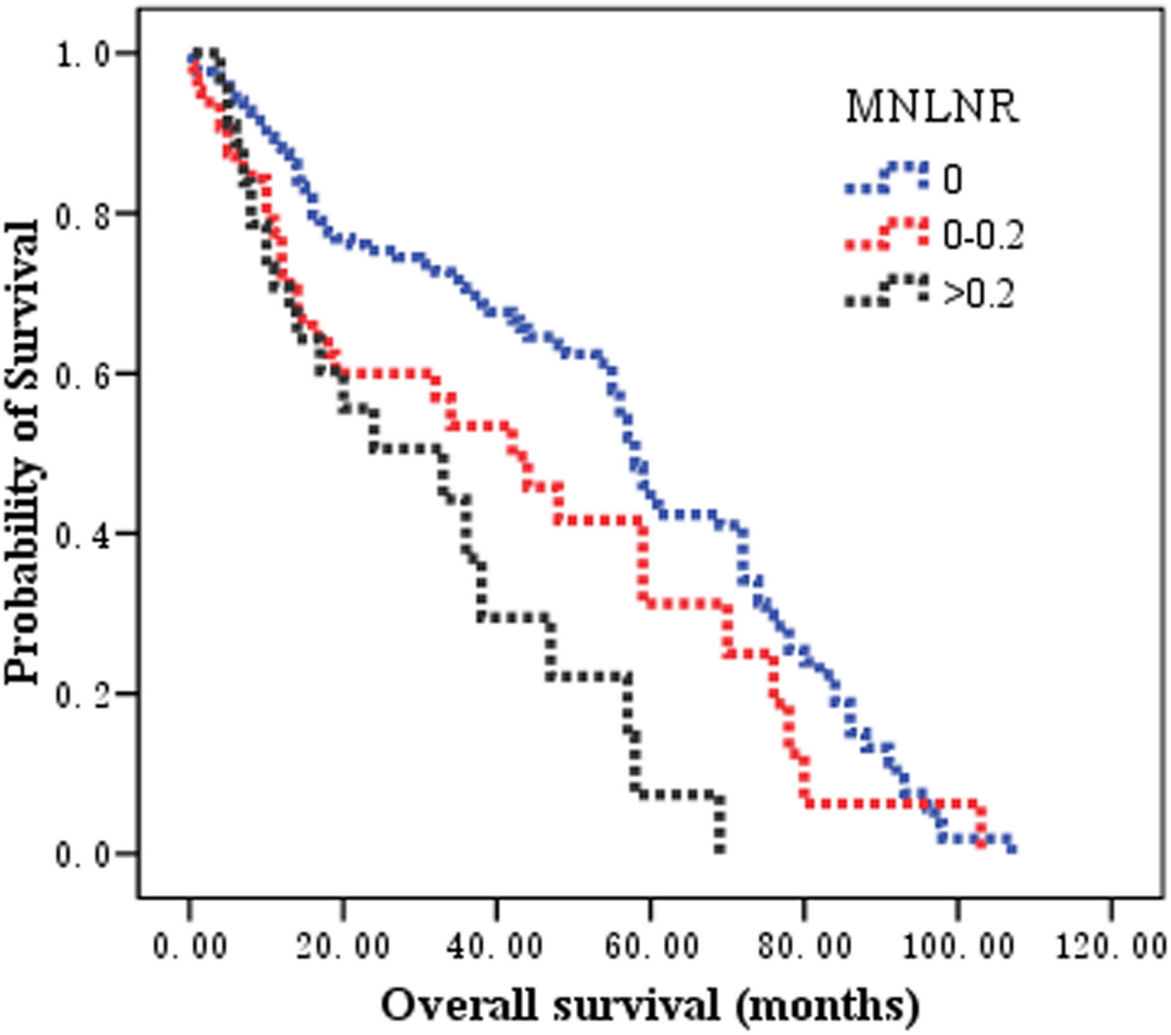
The prognostic significance of MNLNR category on overall survival in patients with esophageal squamous cell carcinoma after esophagectomy Kaplan-Meier survival analysis of MNLNR subgroups by log-rank test (Chi-square = 17.375, *P* = 0.000).

Patients classified as MNLNR 0, MNLNR 0.01-0.2, and MNLNR >0.2, the observed 5-year OS rates were 46.6%, 31.2%, and 7.4%, respectively, and the median survival times of these three groups were 58.0, 42.0, and 33.0 months, respectively (χ2 = 17.375, *P* = 0.000 by log-rank test). The survival curves according to the pN category, the LNR category, and MNLNR are shown in Figure 2, Figure 3 and Figure 4.

The clinicopathological factors analyzed in the univariate survival analysis are also shown in Table 1. The factors significantly influencing the 5-year OS were sex (P = 0.040), histological grade (P= 0.044), pT stage (P = 0.022), pN stage (P = 0.007), LNR category (P = 0.000), and MNLNR (P = 0.000) after esophagectomy.

### Multivariate survival analysis

Multivariate survival analysis was performed with Cox’s proportional hazard regression model to identify the independent factors correlated with prognosis. There were three different lymph node stages, pN, LNR and MNLNR in the present study. First, we put each lymph node stage into multivariate survival analysis to confirm the prognostic value of each lymph node category. Finally, we put all three stages into final analysis to confirm the value of MNLNR compared with pN and LNR category.

When either N category or LNR category or MNLNR was included in the analysis models, it was found to be one of the most significant independent prognostic factors for OS, in addition to gender and pTNM stage (P<0.05 for these parameters). However, the N category and LNR (P > 0.05) no longer significantly predicted survival when the N category, the LNR category, and the MNLNR were simultaneously considered covariates. By comparison, the MNLNR category (P = 0.003) remained as a significant indicator of prognosis (Table 2).

**Table 2:** Multivariate survival analysis of the variables affecting the overall survival

Variables	Multivariate analysis 1			Multivariate analysis 2			Multivariate analysis 3			Multivariate analysis 3		
	HR	95% CI	P value	HR	95% CI	P value	HR	95% CI	P value	HR	95% CI	P value
Gender	0.572	0.343-0.952	0.031	0.576	0.346-0.958	0.034	0.570	0.343-0.949	0.031	0.570	0.343-0.949	0.031
pN	1.316	1.025-1.684	0.032	-	-	-	-	-	-	0.986	0.625-1.525	0.792
pTNM	1.438	1.048-1.974	0.025	1.422	1.043-1.940	0.026	1.417	1.045-1.922	0.025	1.417	1.045-1.922	0.025
LNR	-	-	-	1.410	1.081-1.840	0.011	-	-	-	0.692	0.267-1.789	0.412
MNLNR	-	-	-	-	-	-	1.342	1.108-1.625	0.003	1.342	1.108-1.625	0.003

LNR, metastatic lymph node ratio; MNLNR, the ratio of the number of metastatic to negative lymph node; HR, hazard ratio; CI, confidence interval.

## DISCUSSION

The LNR has been shown to have better prognostic value than pN stage in EC patients [5-15, 19-23]. Greenstein et al. [22] used the SEER database to evaluate the relationship between LNR and survival among 838 esophageal patients with lymph node metastasis. They classified the patients into three groups according to the LNR (≤0.2, 0.21-0.5, and >0.5), and found that LNR can stratify survival better than the pN stage. A recent study included 387 ESCC patients receiving curative esophagectomy showed that compared with N stage, the LNR stage yielded a potential superiority of the prognostic discriminatory ability and exhibited higher accuracy in determining the prognosis of patients with ESCC [14]. Recent studies showed that use of the NLNs may help to provide an accurate prognosis [17–18]. Regional LNs are the most common initial site of EC recurrence, and these nodes harbor micrometastatic disease that cannot be detected readily by standard H-E staining techniques. Theoretically, resecting more LNs or finding more NLNs may reduce the risk of occult lesions and thus increase the survival rate.

The rationalities to propose the MNLNR were based on the following reasons. 1) The ratio of metastatic to negative lymph nodes could reflect two factors, one is the severities of lymph node metastasis (Numerator) and the other is the radicality of lymph node resection (Denominator). With more lymph node metastasis and less lymph node resection, the ratio would be bigger indicating poorer survival; with less lymph node metastasis and more lymph node resection, the ratio would be smaller indicating better prognosis. 2) The pN stage is affected by the number of removed lymph nodes. The positive lymph nodes may be left in the body if only a few lymph nodes were removed during the operation, which leads to an under-staged disease. LNR has been proposed to eliminate the stage migration. Whether MNLNR could address the problems related to the variability of nodal dissection and stage migration is not known. Consistent with previously reported data, our findings revealed that the number of metastatic nodes increased proportionally to the total number of dissected LNs, but the LNR and MNLNR were not correlated with the total number of retrieved LNs. These results demonstrated that MNLNR was not influenced by lymph node resection just like LNR, but pN was influenced by surgical procedure. 3) We established the cut-point for MNLNR on the basis of the statistical significance of OS observed with increasing values of 0.2 intervals as 0, 0.01-0.20, and >0.20 by performing log-rank test. We found that the LNR (Figure 3) and MNLNR (Figure 4) category showed a clear advantage over the pN category (Figure 2). Our study also confirmed that MNLNR as an independent prognostic factor; still need further investigation with a bigger patient population and more detailed subgroups.

In the present study, the MNLNR category was superior to the pN category and LNR because of the following reasons. (i) In univariate analysis, the log-rank χ2 associated with MNLNR (χ2 = 17.375, *P* = 0.000) was larger than that of the AJCC N category (χ2 = 7.372, P = 0.007) and LNR (χ2 = 16.158, *P* = 0.000), indicating a higher statistical significance. (ii) In multivariate analysis, pN, LNR or MNLNR was an independent prognostic factor for OS, respectively. However, the N stage and LNR category lost the significance when all three covariates were put into the multivariate analysis and compared together (Table 2).

Several limitations should be considered in this study. Firstly, this was a retrospective study at a single cancer center. Secondly, there were only three groups based on the cut-off value of MNLNR for the relatively small patient number. It will be necessary to confirm the additional cut-off value especially for patients with MNLNR > 0.2 and to explore the optimal cut-off point. Thirdly, it has been demonstrated that the station of the positive nodes and the number of station removed are important independent prognostic factor affecting long-term survival in patients with EC. However, the relationship between the MNLNR and the location of the positive LNs and the number of station were not investigated in our study. Finally, one of the limitations of this study is the population; maybe other studies with other populations (Africa, America, and Europe) will be needed.

In conclusion, our investigation demonstrated that the MNLNR category may be a potentially convenient and reproducible prognostic variable to reduce stage migration. The MNLNR should be considered as an independent prognostic factor in ESCC patients after curative esophagectomy. In addition, MNLNR showed a better prognostic value than pN stage and LNR category.

## PATIENTS AND METHODS

### Patients

All patients provided written consent for their information and surgical samples to be stored at the Tianjin Medical University Cancer Center used for research. This study was approved by an independent ethics committee at the Cancer Center of Tianjin Medical University. In 2014, the Department of Esophageal Cancer of Tianjin Medical University Cancer Hospital and Institute established a database of esophagectomy cases by performing a retrospective review of patients who attended the institution. The data collected for the database included patient demographics, preoperative symptoms, comorbidities, risk factors, family history, main preoperative examination results, tumor stage and histopathologic features, follow-up and survival data. Chart reviews were performed solely by experienced clinicians and were recorded on standardized abstraction forms. Data can be extracted and analyzed according to the aims of a particular study, and the database was managed in an anonymous way before the authors accessed the data.

Selective criteria included: (a) no neoadjuvant treatment; (b) complete tumor resection; (c) negative incision margins; (d) postoperative histopathologic confirmation of squamous cell carcinoma; and (e) no perioperative mortality; (f) death due to ESCC progression and cancer-related complications; (g) with follow-up data.

### Esophagectomy

All patients included in our study underwent staging with physical examination, cervical ultrasonography, upper gastrointestinal radiography, endoscopy and endoscopic ultrasound, thoracic and abdominal enhanced computed tomography (CT) and/or positron emission tomography (PET) or PET-CT scans. The surgical approach was based on tumor location, tumor stage, and surgeon preference. All patients underwent transthoracic esophagectomy, and two-field or three-field lymph node dissection (if suspicious for cervical lymph node metastasis). Reconstruction was performed with a gastric tube and a thoracic or left cervical esophagogastrostomy. Pathologic stage was determined according to the 7th edition AJCC staging system [24].

### Lymph node classifications

Lymph node metastasis was classified according to the 7th edition AJCC N category based on the number of metastatic lymph nodes: N0, no metastasis; N1, 1∼2 metastatic LNs; N2, 3∼6 metastatic LNs; and N3, ≥7 metastatic LNs. In the present study, our analysis was conducted as follows to determine the appropriate cut-point of LNR and MNLNR that determines the greatest actuarial survival difference among subgroups. Patients without LNs metastasis were initially assigned to one group because their prognoses significantly differed from patients with lymph node metastasis. The intervals of LNR and MNLNR categories were subsequently determined by comparing the OS rates on the basis of an initial interval of 0.2 and then combining the neighborhood survival curves by using the log-rank test [14].

### Follow-up

The patients attended the institution between 2005 and 2008 were retrospectively reviewed. After curative resection, the patients were followed up according to our standard protocol: every three months for the first two years, every six months during the third to the fifth year, and then annually thereafter until death or the last follow-up. Clinical, laboratory, and imaging examinations were performed in each visit. Endoscopic examinations were performed when necessary. The median follow-up period after surgery for the entire cohort was 30 months (range, 3∼108 months). OS was calculated as the time from operation to the date of death or final follow-up.

### Statistical analysis

Statistical analyses were performed using the SPSS software package (SPSS Standard version 18.0; SPSS, Chicago, IL, USA). Statistical analyses included univariate analyses using the χ2 or Fisher’s exact tests for categorical data. Continuous variables were analyzed using ANOVA. Survival analyses were performed using Kaplan-Meier curves with log rank tests for significance. Multivariate survival analyses were performed using the Cox proportional hazard regression model. All statistical tests were two-sided and P value less than 0.05 was considered significant.

The variates for univariate analysis included gender (male or female), age (<65 years or ≥65 years), smoking and drinking history (yes or no), tumor location (upper, middle, lower), tumor size (<4 cm or ≥4 cm), histological grade (well, or moderate and poor differentiation), pT category (T1-2 or T3-4), pN category (N0, N1, N2, or N3), pTNM stage (I, II, III, or IV), LNR category (0, 0.01-0.20, or 0.21-1.0), MNLNR category (0, 0.01-0.20, >0.2). Only those variates with P value less than 0.05 in univariate analysis were underwent multivariate survival analyses.
